# Characterization of PTFE Film on 316L Stainless Steel Deposited through Spin Coating and Its Anticorrosion Performance in Multi Acidic Mediums

**DOI:** 10.3390/ma13020388

**Published:** 2020-01-14

**Authors:** Waseem Akram, Amer Farhan Rafique, Nabeel Maqsood, Afzal Khan, Saeed Badshah, Rafi Ullah Khan

**Affiliations:** 1 Department of Mechanical Engineering, International Islamic University, Islamabad 44000, Pakistan; waseem.akram@iiu.edu.pk (W.A.); rafiullah.khan@iiu.edu.pk (R.U.K.); 2 Department of Aerospace Engineering, Faculty of Engineering, King Abdulaziz University, Jeddah 21589, Saudi Arabia; afrafique@kau.edu.sa; 3 Department of Production Engineering, Faculty of Mechanical Engineering and Design, Kaunas University of Technology, 51424 Kaunas, Lithuania; nabeel.maqsood@ktu.edu; 4 Department of Mechanical Engineering, University of Engineering & Technology, Peshawar 25000, Pakistan; afzalkhan@uetpeshawar.edu.pk

**Keywords:** polytetrafluoroethylene, 316L stainless steel, corrosion performance, coating, microstructure morphology

## Abstract

Polytetrafluoroethylene (PTFE) was coated on 316L stainless steel (SS) substrate through a spin coating technique to enhance its corrosion resistance properties in hydrochloric acid (HCl) and nitric acid (HNO_3_) medium. Scanning electron microscopy (SEM) revealed the morphology of the coated and uncoated substrates and showed a uniform and crack-free PTFE coating on 316L SS substrate, while a damaged surface with thick corrosive layers was observed after the electrochemical test on the uncoated sample. However, an increased concentration of HCl and HNO_3_ slightly affected the surface morphology by covering the corrosive pits. An atomic force microscope (AFM) showed that the average surface roughness on 316L SS and PTFE coating was 26.3 nm and 24.1 nm, respectively. Energy dispersive X-ray spectroscopy (EDS) was used for the compositional analysis, which confirmed the presence of PTFE coating. The micro Vickers hardness test was used to estimate the hardness of 316L SS and PTFE-coated substrate, while the scratch test was used to study the adhesion properties of PTFE coating on 316L SS. The anticorrosion measurements of 316L SS and PTFE-coated substrates were made in various HCl and HNO_3_ solutions by using the electrochemical corrosion test. A comparison of the corrosion performance of PTFE-coated substrate with that of bare 316L SS substrate in HCl medium showed a protection efficiency (PE) of 96.7%, and in the case of HNO_3_ medium, the PE was 99.02%, by slightly shifting the corrosion potential of the coated sample towards the anodic direction.

## 1. Introduction

Stainless steels (SS) are the most commonly used materials in different engineering applications, mainly because of their enhanced mechanical and excellent corrosion resistance properties. These properties also extend their use to making different biomaterials for use in the manufacturing of various medical devices [1,2]. 316L SS has good corrosion resistance in strong reducing media such as dilute sulfuric acid solution and a mixture of acetic acid and formic acid at room temperature. However, as the temperature rises, passivation becomes unstable and the corrosion resistance decreases considerably [3,4,5]. The excellent corrosion resistance of SS is provided by the formation of an oxide layer of Cr_2_O_3_ on the metallic surface of 316L SS [6,7]. Stainless steels lack high temperature corrosion resistance in strongly aggressive media containing Cl^−^ ions [8,9,10] and under the influence of aggressive chloride anion. The local breakdown in passivity occurs mainly at sites of local heterogeneities, causing pitting corrosion [11,12], and corrosion can also occur under high temperatures and high pressure stress [13]. For these reasons, stainless steels may need to be improved by corrosion protection. A number of innovative techniques are currently under intensive study to improve the corrosion behavior of stainless steel, such as sol-gel deposition [14,15], chemical vapor deposition [16], plasma-nitriding [17], plasma detonation techniques [18], high-velocity oxy-fuel spray (HVOF) [19] and atomic layer deposition (ALD) [20,21].

The bomb calorimeter device is a practical tool that can be used to measure the combustion heat of materials and changes in chemical reactions (such as acid-base neutralization, dissolving, solid-state reaction, and crystal phase transition), physical changes (such as melting, evaporation, dehydration) as well as heat capacity. To examine the heat of combustion and calorific value of the propellant and explosive testing of ammonium perchlorate (AP)-based composite propellants, which is used as missile fuel, the propellant is placed in the crucible, which typically contains a multi-modal distribution of AP (NH_4_ClO_4_) grains embedded in the hydroxyl-terminated polybutadiene (HTPB) matrix. These composite propellants, based on ammonium perchlorate (AP) without aluminum, generate reduced smoke, HCl and H_2_O on combustion. Nitric acid (HNO_3_) and sulfuric acid (H_2_SO_4_) will be formed if the sample contains nitrogen and sulfur, respectively.

A number of resistive coatings for corrosion prevention of 316L SS have been developed by using the sol-gel method, including the use of a smooth, uniform, thin film of Titania (TiO_2_) nanoparticles to improve the corrosion resistance [12] and the use of nanometric Al_2_O_3_ and TiO_2_ alternating composite layers on 316L SS substrates deposited via ALD [19]. Corrosion and wear performance have also been improved by the use of NiTi coating modified with ZrO_2_ coatings on AISI 316 SS [22] by using a cold spray technology (a coating process in which a metal substrate is exposed to small particles (1 to 50 μm), which are accelerated by the supersonic flow of compressed gas at a high speed (300–1200 m/s)). The process is based on a combination of particle temperature, speed and size to allow spraying at the lowest possible temperature to: coat the cobalt chromium (Co–Cr) alloy L605 powders in order to improve the corrosion resistance and the strength of 316L SS [23]; Pd–Ni/Pd–Cu double coating on the 316SS surface by electroplating [24]; nanocomposite coatings of polychlorotrifluoroethylene (PCTFE) particles with nickel–tungsten (Ni–W) coatings through the electro deposition procedure [25]; polymerized vinyltrimethoxysilane (PVTMS) coating with henna’s aqueous solution forming a PVTMS/henna hybrid sol-gel for biomedical applications [26]; polyaniline-graphene nanocomposite coatings electrodeposited on 310 SS by cyclic voltammetry technique [27]; and synthesis of polyaniline-montmorrilonite nanocomposite coatings deposited on 316L SS by electrodeposition [28,29].

The currently available literature reveals that previous studies have mostly focused on developing a corrosion resistive coating in salt solutions and/or weak HCl mediums. The objective of this research is to develop a corrosion resistive coating for use in strong HCl and HNO_3_ environments.

Polytetrafluoroethylene (PTFE) is a polymer that has attracted significant interest and is of considerable importance due to its extensive applications. It is characterized by its chemical inertness and hydrophobic, antifriction, nonstick and self-lubricating properties coupled with its high temperature resistance (melting temperature of 325 °C) [30,31,32,33].

The aim of this research was to deposit a PTFE coating on 316L SS and to study its anticorrosion performance in the HCl and HNO_3_ mediums. The work also investigated the surface morphology of the coatings before and after the electrochemical corrosion test.

The current research aimed to develop a resistive coating of SS in HCl. In the present paper, a uniform coating of PTFE was successfully applied to 316L SS substrate using a spin coating technique and then we investigated the corrosion performance in HCl and HNO_3_ solutions of various concentration, which has not been studied yet. The electrochemical corrosion test and potentiodynamic polarization test were employed to measure the anticorrosion performance of the PTFE coating, and to discuss the mechanism of corrosion resistance. The morphology, compositional and surface roughness of the coating were studied through SEM, EDX and AFM, respectively.

## 2. Materials and Methods

### 2.1. Sample Preparation

The 316L SS samples were separated from the sheet and cut into square specimens with dimensions of 16.55 mm × 16.55 mm × 1 mm and were used as substrates. Before the coating process, the substrates were mechanically polished with 80–2000 grit sized SiC paper and rinsed with distilled water. Then the substrates were sonicated in acetone to remove residual grease and washed with distilled water and finally dried at room temperature overnight. PTFE was obtained commercially from DuPont and used without pre-treatment. Table 1 shows the chemical composition of 316L stainless steel.

### 2.2. Coating of PTFE on 316L SS

Spin coating was used to deposit a PTFE thin film on 316L SS substrates. Spin coating is a procedure in which a small amount of fluid is deposited drop-wise on to the center of the substrate while it is spinning at a high speed (3000 rpm). Centripetal acceleration will allow it to spread to the edges of the substrate, forming a thin layer on the substrate’s surface. The coatings were obtained at room temperature using a spin rate of 3000 rpm, and 3–5 drops of PTFE were deposited drop-wise on to the center of the substrate, leaving a uniform layer. The coated substrates were dried temporarily on a hot plate at 100 °C for 15 min and then the samples were further annealed at 200 °C in a vacuum oven for 2 h to reduce the stresses and relax the PTFE film on to the SS.

### 2.3. Characterization of PTFE Coating on 316L SS Substrate

The surface morphology of PTFE coating and uncoated substrates were studied through JSM5910 JEOL scanning electron microscope (SEM, JEOL, Akishima, Tokyo, Japan) and the compositional analysis was performed with an energy dispersive X-ray spectroscopy (EDS) equipped with SEM. The changes in the surface morphologies of the coated and uncoated substrates were also studied and compared after the electrochemical corrosion test in both the solutions with various concentrations. The thickness of the coating was measured with SEM. The surface roughness of the coated and uncoated substrates was estimated through JSPM 5200 atomic force microscopy (AFM, JEOL, Akishima, Tokyo, Japan).

### 2.4. Electrochemical Corrosion Test

Electrochemical and potentiodynamic polarization tests were performed using the Gamry framework instrument MULTECHEM SERIES G750 (Warminster, PA, USA) to evaluate the corrosion of 316L SS in 13.05 M HCl solution and PTFE coating in 6.5 M, 9.79 M and 13.05 M HCl solutions. Similarly, the same instrument was used to evaluate the corrosion measurement of 316L SS in 9.58 M HNO_3_ solution and PTFE-coated substrates in 4.79 M, 7.189 M and 9.58 M HNO_3_ solutions.

All the experiments were carried out using a three-electrode corrosion cell setup at room temperature with a coated sample of 1 cm^2^ exposed area as the working electrode, a saturated calomel electrode (SCE) as reference and graphite as the counter electrode. HCl solution was used as a corrosive medium prepared with distilled water. Polarization measurements were carried out using a scan rate of 1 mV/s at a potential initiated at −500 mV to +300 mV versus corrosion potential after an initial 60 min exposure to the test electrolyte to achieve a stabilized open circuit potential. Corrosion potential (*E_corr_*), corrosion current density (*i_corr_*) and corrosion rate (C.R) were determined using the Tafel diagram and potentiodynamic polarization curves by taking the intersection point of the anodic and cathodic curves. In order to create a better means of comparison, corrosion rates were calculated according to ASTMG 102-89 [21] using equation.

(1) $$C.R=1.288\;\times \;10^{-5}\;\times \;i_{corr}\;\times \;\frac{E.W}{\rho }$$

where *C.R* is the corrosion rate in mm per year (mpy), *i_corr_* is the corrosion current density (μA/cm^2^), EW is the equivalent weight, and ρ is the material density (g/cm^3^).

From the measured *i_corr_* values, the protection efficiency (PE) was obtained from the following equation [34].

(2) $$PE=\frac{\left(i_{corr}-i_{corr\left(c\right)}\right)}{i_{corr}}\;\times \;100$$

where *i_corr_* and *i_corr(c)_* are the corrosion current density values in the absence and presence of the coating, respectively.

### 2.5. Mechanical Testing

The micro hardness of 316L SS and PTFE-coated substrate was performed on the sample surface using a load of 300g for 15 s using a HVS-1000 micro hardness tester (Jinan Victory Instrument Co., Ltd., Jinan, China) on the Vickers scale with a diamond square-base pyramid with an angle of 136° at the vertex between opposite sides. Samples were adequately refined to confirm that the micro-hardness measurements were accurate.

A mechanized scratch tester 705 (TQC Sheen, Capelle aan den Ijssel, The Netherlands) was used to evaluate the adhesion properties of the coatings based on the technique of scratching resistance using the ISO 1518-1 standard [35] at room temperature. Variable loads were applied to achieve various degrees of failure from trace to destruction. A voltmeter was then attached to the front panel, which shows the connection of tip of the tool with the substrate of metallic sample. To observe any sign of penetration in the coating surface, a test panel of the area was subjected to scratching. A magnifying glass was used as a gauge to measure the scratch. Load was continuously applied until the desired results were achieved, as the weight increments must be between 100–2000 g. When the needle on the meter swings over to the right-hand side, it indicates the complete coating failure of the metallic test panels. According to the respective standard, the diameter of the needle was a 1 mm hemispherical tip stylus.

## 3. Results and Discussion

### 3.1. Characterization

**Figure 1:**
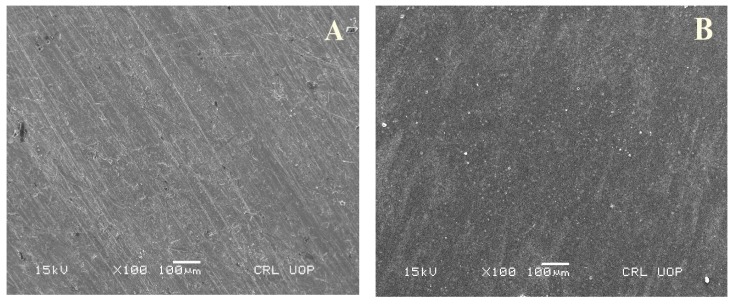
SEM micrographs of (**A**) uncoated substrate and (**B**) polytetrafluoroethylene (PTFE)-coated substrate.

The surface morphology of the bare and coated substrate was analyzed by the SEM. Figure 1 shows the surface morphology of uncoated substrate and PTFE-coated substrate.

**Figure 2:**
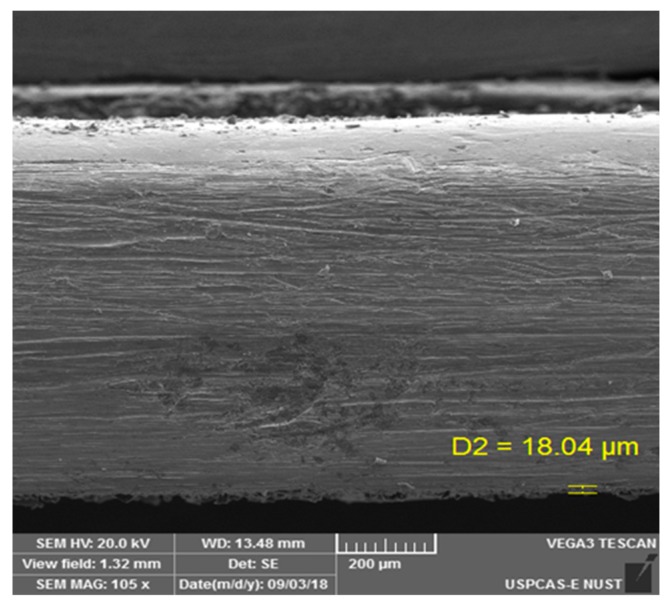
Cross section of the PTFE coating on 316L SS.

The thickness of the coating measured with SEM was approximately 20 µm. Figure 2 shows the cross section of the coating.

**Figure 3:**
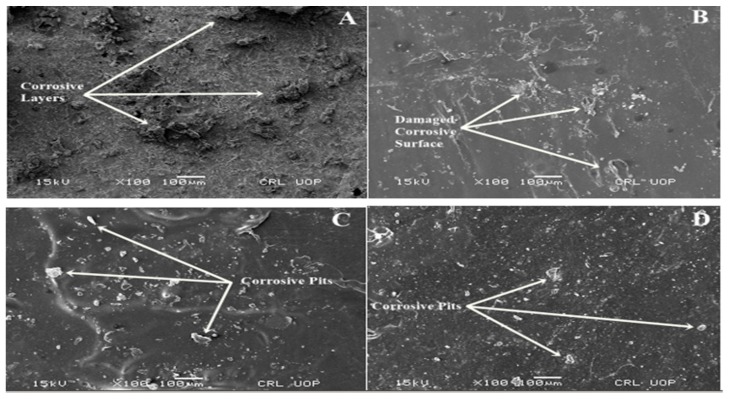
SEM micrograph after the electrochemical corrosion test of (**A**) uncoated substrate in 13.05 M HCl solution; (**B**) PTFE-coated substrate in 6.5 M HCl solution; (**C**) PTFE-coated substrate in 9.79 M HCl solution; (**D**) PTFE-coated substrate in 13.05 M HCl solution.

Figure 3 gives the SEM micrographs after the electrochemical corrosion test of uncoated substrate in a 13.05 M HCl medium, and PTFE-coated substrate in 6.5 M, 9.79 M and 13.05 M HCl medium, respectively.

**Figure 4:**
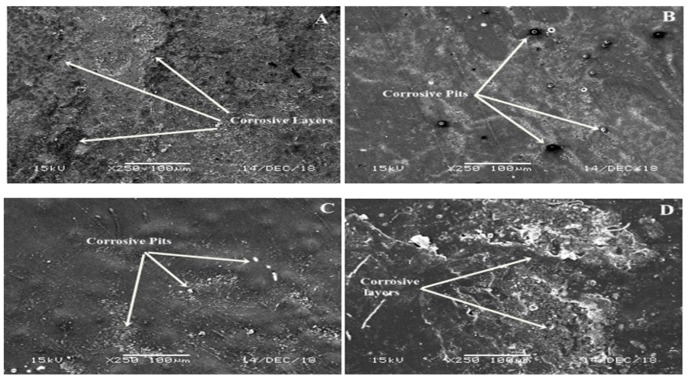
SEM micrograph after the electrochemical corrosion test of (**A**) uncoated substrate in 9.58 M HNO_3_ solution; (**B**) PTFE-coated substrate in 4.79 M HNO_3_ solution; (**C**) PTFE-coated substrate in 7.19 M HNO_3_ solution; (**D**) PTFE-coated substrate in 9.58 M HNO_3_ solution.

The micrographs of the uncoated substrate show some micro cracks on the surface of the substrate while the PTFE coating (Figure 1) shows a uniform and smooth distributed layer on the SS with no discontinuous deposition or cracks observed by SEM, which is in line with previously reported studies [36,37]. PTFE nanoparticles can fill the micropores, and a compact, smooth and regular surface morphology can be obtained, which generally decreases the surface roughness [38,39]. Damage on the surface can be observed in Figure 3A after the electrochemical test on the uncoated sample; it shows destructive and thick layers of corrosion over the surface of the substrate. In comparison, the coated substrates (Figure 3B–D) showed some surface pits (white spots), revealing a more protected surface. PTFE coatings after the electrochemical corrosion test in 6.5 M HCl medium showed little damage, however, an increase in the HCl concentration from 9.79 M to 13.05 M HCl slightly affected the surface morphology by covering the corrosive pits. The presence of small pits on the surface morphology can be clearly seen on coated samples after the electrochemical corrosion tests, but the surface still remained protected in comparison to the uncoated substrate, thus confirming their potential to remain protected in strong HCl environments. Figure 4 shows the SEM micrographs taken after the electrochemical corrosion test, of uncoated substrate in a 9.58 M HNO_3_ solution and PTFE-coated substrate in 4.79 M, 7.19 M and 9.58 M HNO_3_ medium, respectively. The comparison shows the result of changes in the morphology of the PTFE coating after the corrosion test. In Figure 4A, the corrosive layer over the surface of the uncoated substrate can be clearly seen and the thick corrosive layer shows the damaged morphology of the unprotected surface in a 9.58 M HNO_3_ solution. In comparison with the coated substrates, it shows more protected surface, indicating that there is not much affected surface. In contrast, coated substrates after electrochemical corrosion tests in 4.79 M, 7.19 M and 9.58 M HNO_3_ medium show protection. The presence of small pits on the surface morphology can be clearly seen on coated samples after electrochemical corrosion tests but remain protected compared to uncoated substrate in strong HNO_3_ medium. In a previous study [36], PTFE/Ni–B coated steel was immersed in 3.5% NaCl solution for different times, and images showed that the untreated steel became corroded after a few days but no changes occurred in the PTFE/Ni–B coated steel after 7 days immersion in 3.5% NaCl solution.

**Figure 5:**
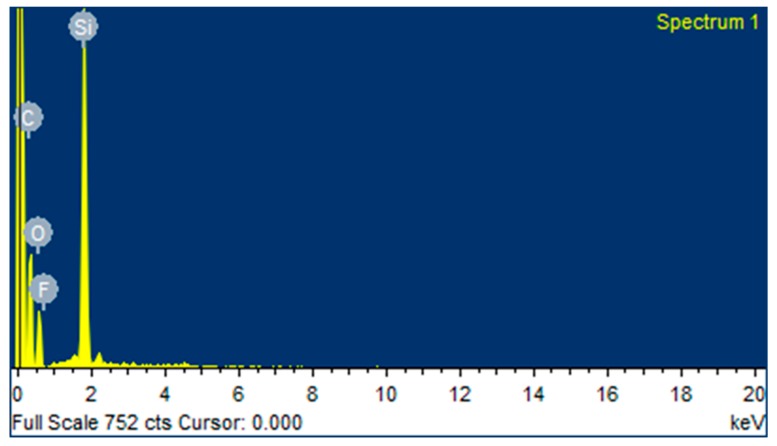
Energy dispersive X-ray spectroscopy (EDS) peaks of PTFE coating.

**Figure 6:**
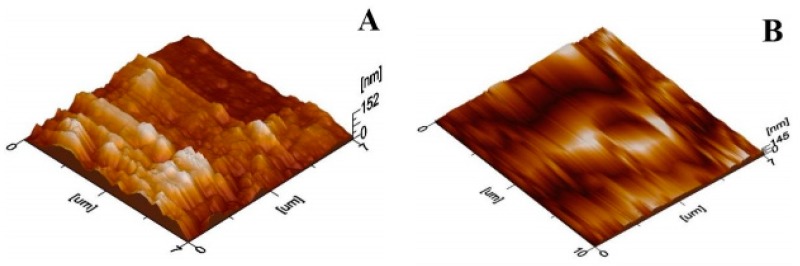
Atomic force microscope (AFM) images of (**A**) uncoated and (**B**) coated substrates.

The compositional analysis of PTFE coating was performed with EDS and the results are shown in Figure 5 and Table 2, which reveal the peaks and elemental compositions of PTFE, respectively. Figure 5 shows the peaks of carbon (C), oxygen (O), fluorine (F) and silicon (Si), which confirms the presence of PTFE coating. The above -mentioned author used the EDS analysis of Ni–W/PTFE coating to confirm the presence of C and F peaks [36]. The AFM (Figure 6) of uncoated 316L SS showed an average surface roughness value of 26.3 nm whereas the coated sample showed a lesser surface roughness of 24.1 nm, further suggesting that an increased PTFE particle presence will decrease the surface roughness [33].

### 3.2. Corrosion Measurement

**Figure 7:**
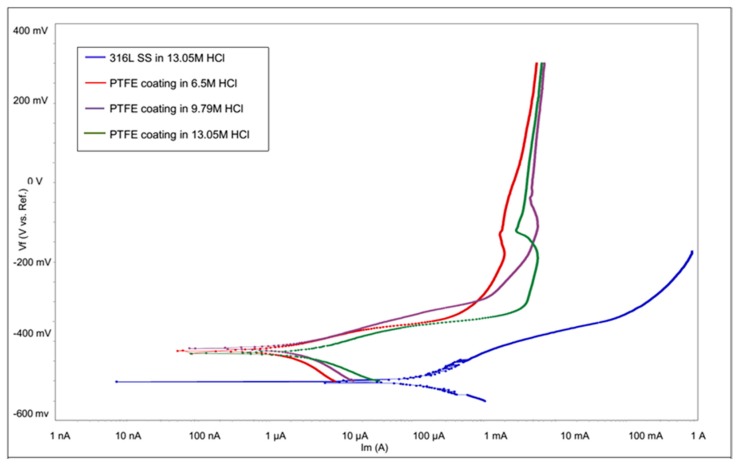
Potentiodynamic polarization curve of 316L SS and PTFE coating in HCL.

The values of *E_corr_* and *i_corr_* were calculated by using the Gamry framework software (MULTECHEM SERIES G750). The electrochemical corrosion test was performed on the uncoated 316L SS in 13.05 M HCl solution and PTFE-coated sample in the 6.5 M, 9.79 M and 13.05 M HCl solutions after 60 min exposure to the solution. The potentiodynamic polarization curves of uncoated 316L SS and PTFE-coated samples tested in HCl medium are shown in Figure 7.

It can be seen that 316L SS exhibited an average *E_corr_* of −502.559 mV, *i_corr_* of 80.76 µA/cm^2^ and *C.R* of 29.55735 mpy (milli inch per year), which indicates very poor corrosion resistance. However, PTFE-coated substrate tested in 6.5 M HCl exhibited an *E_corr_* of −424.659 mV, *i_corr_* of 1.065 µA/cm^2^ and *C.R* of 0.3896725 mpy, when the concentration of HCl increased to 9.79 M, it exhibited an *E_corr_* of −417.659 mV, *i_corr_* of 1.791 µA/cm^2^ and *C.R* of 0.655474 mpy. Similarly, when the concentration increased to 13.05 M, the *E_corr_* shifted to −431.559 mV, *i_corr_* to 2.672 µA/cm^2^ and *C.R* of 0.9779258 mpy. Electrochemical data for the corrosion test in HCl solution are listed in Table 3.

Firstly, the *C.R* of 316L SS substrate and PTFE coating tested in 13.05 M HCl solution was compared, and coated substrate was found to be much nobler. Previously [35], the *i_corr_* of PTFE/PEO coating was measured as 4.93 × 10^−2^ µA/cm^2^ in 3.5 wt.% NaCl solution. The comparison of bare and PTFE-coated samples showed a PE of 96.7% providing superior corrosion protection. The bare 316L SS substrate *C.R* value was observed to be very high due to the unprotected surface layer, which led the hydrochloric acid to dissolve the oxides, especially in the initial stage where the rate of atoms collision is very high. Moreover, the corrosion potential of the coated sample was slightly shifted toward the anodic direction by increasing concentration. Furthermore, in a previous study [36], the author claimed that adding 40% PTFE to Ni–B coated steel increases the *E_corr_* and reduces *i_corr_* compared to Ni–B coated steel.

**Figure 8:**
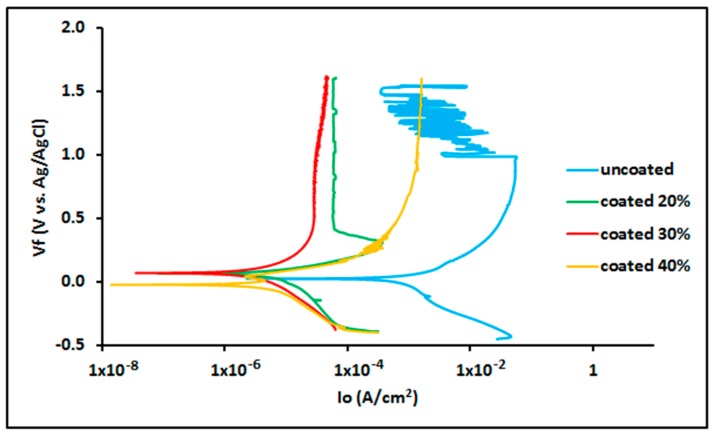
Potentiodynamic polarization curve of 316L SS and PTFE coating in HNO_3_.

There are similarities in the case of HNO_3_ medium solution. The potentiodynamic polarization curves of uncoated 316L SS and PTFE-coated samples tested in HNO_3_ medium are shown in Figure 8.

The electrochemical data and results of the polarization test performed on 316L SS and PTFE-coated substrate are shown in Table 4, where it can be seen that the 316L SS substrate possessed an *E_corr_* of 26.80 mV, *i_corr_* of 1480 µA/cm^2^ and *C.R* of 684.8 mpy in 9.58 M HNO_3_ solution, which shows even worse corrosion resistance when compared to HCl medium.

However, the application of PTFE coating resulted in superior performance and exhibited an *E_corr_* of 67.60 mV, *i_corr_* of 8.840 µA/cm^2^ and *C.R* of 4.080 mpy in 4.79 M HNO_3_ solution in 6.5 M HCl solution, an *E_corr_* of 71.30 mV, *i_corr_* of 9.010 µA/cm^2^ and *C.R* of 4.157 mpy in 7.19 M HNO_3_, and an *E_corr_* of −20.80 mV, *i_corr_* of 14.50 µA/cm^2^ and *C.R* of 6.676 mpy in 9.58 M HNO_3_ solution. Thus, the comparison of the bare 316L SS substrate with that of PTFE-coated material in 9.58 M HCl solution shows a PE of 99.02%. The polymer coating covers the cracks and holes and the self-lubricating PTFE polymer layer enhances the anticorrosion properties [36].

**Figure 9:**
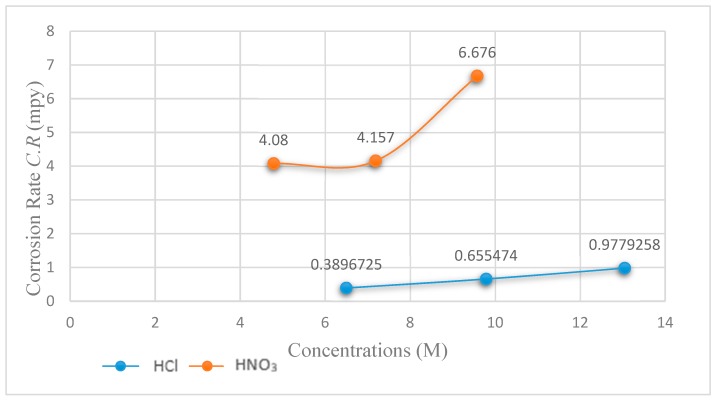
Graph of the corrosion rate (C.R) of coated substrates vs. HCl and HNO_3_ concentrations.

Figure 9 shows the graph of *C.R* versus HCl and HNO_3_ concentrations of the coated substrates. It can be seen from the comparison that *C.R* significantly increased to 68.2% when the HCl concentration increased from 6.5 M to 9.79 M while increasing the HCl concentration from 9.79 M to 13.05 M resulted in a 49.2% increase in the C.R. Similarly, the *C.R* increased by 1.88% when the HNO_3_ concentration shifted from 4.79 M to 7.19 M while shifting the HNO_3_ concentration from 7.19 M to 9.58 M resulted in a significant increase in the *C.R* by 60.59%. As a result, the corrosion potential of PTFE coating shifted positively when the corrosion current density was lowered compared to the uncoated 316L SS, signifying it has much better corrosion resistance.

### 3.3. Mechanical Testing

**Figure 10:**
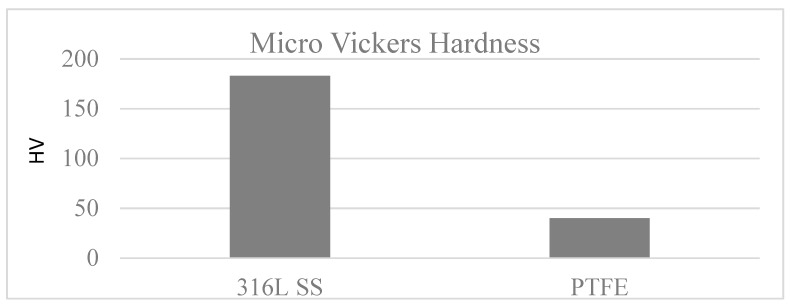
Micro Vickers hardness of 316L SS and PTFE coating.

The Vickers micro hardness test on 316L SS and PTFE-coated samples was performed at five different positions and an average of the measurements was reported. The 316L SS substrate showed a relatively high average hardness value of 183 HV (Figure 10), in comparison the PTFE coating was found to be soft due to its polymeric nature [40] with an average hardness value of 40 HV.

**Figure 11:**
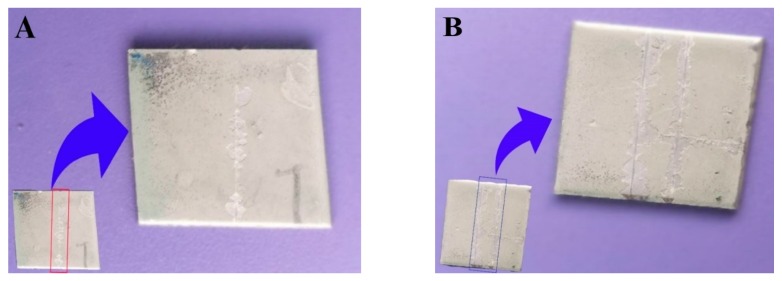
Scratch test result of (**A**) initial scratch on 500 g and (**B**) scratch to substrate on 800 g.

At the initial stage of the scratch test, the load applied on the sample was 100 g, 200 g and 300 g but no scratch was found on the 316L SS PTFE-coated sample because there was no deflection in the voltage needle. After increasing the loading to 500 g on the coated sample, a minor scratch was detected on the sample but with a loading of 800 g, a desirable scratch was observed as the coating was damaged and the voltage needle flicked over, which shows that a short circuit occurred. Figure 11 shows the result of the initial 500 g test and the 800 g test, respectively.

## 4. Conclusions

1. A uniform PTFE coating was successfully deposited on 316L SS substrate by using the spin coating technique. Morphology results showed a smooth surface with no cracks or discontinuations in the coated substrate. SEM and electrochemical corrosion tests revealed the enhanced damage to the bare substrate with thick corrosive layers compared to the coated substrate, in which some surface pores or corrosive pits were detected on the surface, revealing more protected surface after the electrochemical test in the HCl and HNO_3_ medium. However, an increase in the concentration slightly affected the surface morphology by covering the corrosive pits.
2. The comparison of the corrosion performance of PTFE-coated substrate with that of the bare 316L SS substrate in 13.05 M HCl solution at room temperature showed a PE of 96.7%, while in 9.58 M HNO_3_ solution the PE was 99.02%. This revealed that PTFE coating on SS provided superior corrosion resistance. The results showed a remarkable refinement in the corrosion resistance of PTFE coating by decreasing the corrosion rate and corrosion current density in both of the mediums. Thus, PTFE coating shows great potential to be used in harsh acidic industrial environments where high corrosion resistance is required.
3. AFM results showed that the 316L SS and PTFE coating had an average surface roughness value of 26.3 nm and of 24.1 nm, respectively. EDS study confirmed the presence of C and F peaks in the coating.
4. The micro Vickers hardness test revealed that the 316L SS substrate have a relatively high hardness value of 183 HV while the PTFE coating was found to be soft with a hardness value of 40 HV. Scratch tests revealed that it can bear a load up to 500 g, but after that a detectable scratch was observed. Thus, this work suggests the use of PTFE coatings in a severe, acidic environment where high corrosion resistance is required.

## Figures and Tables

**Table 1:** Chemical composition of 316L stainless steel (wt.%).

Elements	C	Mn	P	S	Si	Cr	Ni	Mo	Fe
**Compositions (wt.%)**	0.03	2.00	0.045	0.03	0.75	17.4	12.58	2.28	Bal.

**Table 2:** Elemental compositions of PTFE.

Elements	C	O	F	Si
**Compositions (wt.%)**	21.25	13.70	10.23	54.82

**Table 3:** Electrochemical data of 316L SS and PTFE coating in HCl.

Specimen	HCl Concentration	*E_corr_* (mV)	*i_corr_* (µA/cm^2^)	*C.R* (mpy)
316L SS	13.05 M	−502.559	80.76	29.55735
PTFE coating	6.5 M	−424.659	1.065	0.3896725
PTFE coating	9.79 M	−417.659	1.791	0.655474
PTFE coating	13.05 M	−431.559	2.672	0.9779258

**Table 4:** Electrochemical data for 316L SS and PTFE coating in HNO_3_.

Specimen	HNO_3_ Concentration	*E_corr_* (mV)	*i_corr_* (µA/cm^2^)	*C.R* (mpy)
316L SS	9.58 M	26.80	1480	684.8
PTFE coating	4.79 M	67.60	8.840	4.080
PTFE coating	7.19 M	71.30	9.010	4.157
PTFE coating	9.58 M	−20.80	14.50	6.676
